# Disrupted Brain Network in Children with Autism Spectrum Disorder

**DOI:** 10.1038/s41598-017-16440-z

**Published:** 2017-11-24

**Authors:** Ke Zeng, Jiannan Kang, Gaoxiang Ouyang, Jingqing Li, Junxia Han, Yao Wang, Estate M. Sokhadze, Manuel F. Casanova, Xiaoli Li

**Affiliations:** 1 State Key Laboratory of Cognitive Neuroscience and Learning & IDG/McGovern Institute for Brain Research, Beijing Normal University, Beijing, 100875 China; 2 College of Electronic & Information Engineering, Hebei University, Baoding, 071002 China; 3 Institute of Electrical Engineering, Yanshan University, Qinhuangdao, China; 4 Department of Biomedical Sciences, University of South Carolina School of Medicine Greenville Campus, Greenville Health System, Greenville, SC USA

## Abstract

Alterations in brain connectivity have been extensively reported in autism spectrum disorder (ASD), while their effects on the topology of brain network are still unclear. This study investigated whether and how the brain networks in children with ASD were abnormally organized with resting state EEG. Temporal synchronization analysis was first applied to capture the aberrant brain connectivity. Then brain network topology was characterized by three graph analysis methods including the commonly-used weighted and binary graph, as well as minimum spanning tree (MST). Whole brain connectivity in ASD group was found to be significantly reduced in theta and alpha band compared to typically development children (TD). Weighted graph found significantly decreased path length together with marginally significantly decreased clustering coefficient in ASD in alpha band, indicating a loss of small-world architecture to a random network. Such abnormal network topology was also demonstrated in the binary graph. In MST analysis, children with ASD showed a significant lower leaf fractions with a decrease trend of tree hierarchy in the alpha band, suggesting a shift towards line-like decentralized organization in ASD. The altered brain network may offer an insight into the underlying pathology of ASD and possibly serve as a biomarker that may aid in diagnosis of ASD.

## Introduction

Recent epidemiological studies have reported that nearly 1 in 88 children has some form of autism spectrum disorder (ASD), which is much higher than the expected^1^. ASD is a neurodevelopmental disorder characterized by communication difficulties and social deficits, stereotyped and repetitive behavior, and cognitive delays in some cases. Most individuals with ASD suffer from educational, medical, and social difficulties that have a serious negative effect on their quality of life^2^. In recent years, with the societal awareness of ASD heightening, there is a substantial growth in the related research. However, progress in identifying the underlying neuropsychological and patho-physiological mechanisms is limited. There is a widespread hope for exploring valid biomarkers that can reveal clues to the cause(s) of ASD and enable earlier and more targeted methods for diagnosis and intervention^3^.

It is widely acknowledged that ASD is a disorder of brain development thus prompting an increasing number of studies adopting brain imaging techniques to understand the neurobiology of this condition. Electroencephalography (EEG), directly measuring postsynaptic brain activity in the neocortex, holds both practical and theoretical advantages directed at elucidating clinical biomarker in neurodevelopmental disorders such as ASD^4^. Compared with functional Magnetic Resonance Imaging (fMRI), EEG has higher temporal resolution and higher relative tolerance for movement. Besides, EEG is non-invasive, unlike positron emission tomography (PET), and can be used to collect repeated measurements. The most meaningful is that EEG is clinically available as a cost-effective tool for mass screening of ASD populations and monitoring treatment outcomes^4,5^. Particularly, resting-state EEG (rsEEG) is widely used for studying the children with ASD, this is because rsEEG does not require subject to make a response, which is crucial for patients who can’t perform cognitive tasks accurately.

It has been demonstrated that cerebral EEG can characterize neural system abnormalities associated with ASD^6,7^. Power analysis of rsEEG in ASD reveals a U-shaped profile of power alterations with excessive power in low and high frequency bands^6^. The nonlinear complexity analysis of EEG data indicate a reduction of intrinsic complexity of brain activity in the ASD group at occipital and parietal regions^8^. The atypical EEG complexity can even be used as a biomarker to distinguish infants at high risk for ASD from typically development^9^. It is noted that most of the cognitive and executive functions are based on the coordination and interaction among a large number of neurons that are distributed within and across multiple brain areas, thus emerging models and theories suggest brain activity in functional network, rather than only in specific regions, as being dysfunctional in ASD^10,11^. A large case study with 984 children (430 ASD and 554 Controls) demonstrated when EEG coherence between brain regions were used to identify children with ASD, the classification accuracy can reach up to 86%, suggesting a stable pattern of abnormal functional connection in ASD^12^. Hence, the brain network analysis may be a promising method to reveal the abnormalities associated with ASD.

The interactions (also known as functional connectivity) between brain regions can be estimated by a statistical synchronization between corresponding EEG signals. By computing the synchronization between brain regions, a brain functional network can be constructed. Previous studies have found that alternations of the strength of brain connectivity in ASD are correlated with their behavior features^10,13^. However, whether and how the whole topology of brain networks are affected by abnormal functional connectivity remains largely unclear. In addition, the existing methods based on connectivity are generally too descriptive and lack of a robust framework to discriminate brain networks in different conditions^14^. At present, it is noted that the graph theory can provide an insight in the communication efficiency and general organization of networks, so it has been extensively used to study the organization of the brain network^15,16^. It has been evidenced that brain networks show a small-world organization that owns optimal properties such as high clustering in ordered network and short path length in random network^17^. Such an organization of brain network plays a crucial role in keeping a good balance between global integration (short path) and local specialization (high clustering). Several studies have demonstrated that better cognitive capabilities are associated with higher clustering and shorter paths, while deviant brain topologies may lead to neurological diseases^18,19^. In addition, there is evidence of large scale network reorganization during normal development of brain^20^. Hence, all of these make the investigation of the brain network organization in ASD of great relevance.

A critical point in graph analysis is the comparability of the brain network across different conditions^19^. To increase comparability, there is often a need to normalize the brain networks, such as comparing the network with random networks (in weighed graph analysis), and setting a fixed threshold or link density (in binary graph analysis). However, the normalization would inevitably lead to high sensitivity to alterations in connection strength (for weighted graph analysis) or link density (for binary graph analysis)^21,22^. It has recently been proposed that a so-called minimum spanning tree (MST) may be as an alternative method that avoids these limitations^23^. The MST is defined as an acyclic sub-network of original weighted network that connects all nodes while minimizing connection cost (sum of all connection distances). In this way, all networks in different conditions would have the same number of nodes and edges, enabling a direct comparison of their network topology while avoiding potential biases introduced by the normalization. According to the definition, the MST includes a large proportion of important functional connections and represents the critical backbone of brain network, meaning that it can capture most of the information flow in the original network^24^. Although characterizing brain network with the MST is a matter of ongoing investigation, it has been shown that the MST parameters are altered in neurodegenerative disorder, such as Parkinson’s disease^25^ and Alzheimer’s disease^26^. More recently, the MST method was successfully applied to investigate brain maturation with resting-state EEG^27^. And the study found that the MST can capture neurodevelopmental changes from brain network topology. Hence, the MST may provide a method that is sensitive enough to capture subtle variations in the organization of neural network in ASD.

Studies of brain network in ASD have largely been limited to adults and research earlier in life is lacking^28^. However, identifying the abnormal characteristics during childhood would be more meaningful as it would enable early interventions to these patients. The object of this study is to investigate whether and how resting-state brain networks are abnormally organized in children with ASD. To this end, we recorded resting-state EEG from 21 children with ASD between the ages of 7 and 13 years and 21 age-, gender-, and IQ-matched typically development children. In this study, whole-brain functional networks were constructed in three different methods, including the commonly used weighted and binary graph, as well as MST. Finally, several measures were used to assess the organization of brain networks, including the clustering coefficient and path length for weighted and binary graph, and tree hierarchy and leaf fraction for MST.

## Materials and Methods

### Participants

Twenty-one children diagnosed with high-functioning ASD and 21 age-, gender-, and IQ-matched typically development (TD) children participated in this study. The children with ASD (18 boys, 3 girls) ranged in age from 7 to 13 years (age = 9.9 ± 1.5) with an IQ range of 79 to 134 (IQ = 103.4 ± 16.2); and the TD children (18 boys, 3 girls) ranged in age from 8 to 12 years (age = 10.1 ± 1.3) with an IQ range of 82 to 139 (mean IQ: 106.5 ± 13.4). All children were required to have a Full Scale IQ ≥ 75, as measured by the Wechsler Abbreviated Scale of Intelligence (WASI)^29^.

The ASD participants were recruited from Beijing Haidian Special Education School, and confirmed using DSM-IV-TR criteria (Association 2000) and expert clinical opinion. All the autistic participants met criteria for autism on the two assessments. Potential autistic participants were excluded if they had an associated disorder such as fragile X syndrome and tuberous sclerosis. Potential autistic participants were also excluded in the presence of evidence of head injury or a seizure disorder. Exclusions were based on neurological history and examination. The TD children were volunteers in regular primary school recruited to match the autistic participants on age, Full Scale IQ, gender, and race. Exclusion criteria for typically participants were similar with ASD, with the addition of a family history of an ASD. All participants were right-handed and Chinese.

There were no statistically reliable differences between the ASD and TD participants in age, gender or IQ. Though there exist uneven gender distributions in both groups (only three girls in each group), but the dataset in girls were not statistically different from the boys’. In addition, when these girls were excluded, all of the reported group differences still remain unchanged. Hence, this study included their data. The study protocol received approval from the ethics committee of Beijing Normal University. Written informed consent was obtained from all parents before the start of the experiment. All protocols of the study conform to Declaration of Helsinki guidelines.

### EEG recording and pre-processing

EEG data were acquired with a high-density 128-channel Electrical Geodesics Incorporated system. Electrode impedances were kept below 50 kΩ according to EGI guidelines. The reference electrode was set as the central sensor (Cz), and the sampling rate was 1000 Hz. The subjects were sitting on a comfortable chair during the recording, and they were instructed to keep relaxed with their eyes open for at least 10 min.

For further off-line processing, the original EEG data were re-referenced with the average signal in left and right mastoid sensors as they recorded less signal from the brain^30^. To improve computing speed, EEG data were then down-sampled to 250 Hz. The line noise in EEG was removed by a notch filter centered at 50 Hz. And artifacts (such as electrooculogram and electromyography) in EEG were removed using an EEMD-ICA approach^31^. In addition, visual inspection was performed to reject data segments contaminated with noise. The EEG is typically described in terms of rhythmic activity and the rhythmic activity is divided into bands by frequency. According to the majority of EEG used in clinical practice^32^, artifact-free EEG data were filtered to five frequency bands of interest: delta (1–4 Hz), theta (4–8 Hz), alpha (8–13 Hz), beta (13–30 Hz), and gamma (30–48 Hz). And all the further analyses were performed for the five filtered signals, respectively. The aforementioned pre-processing were conducted with EEGLAB^33^ and self-produced software developed using MATLAB.

### Functional connectivity analysis

This study used phase lag index (PLI)^34^ to compute the synchronization between EEG signals. The PLI is defined as an asymmetry index for the distribution of phase differences between two signals. The asymmetry indicates presence of a consistent lag between two signals. If there is no synchronization, the distribution will be flat. And any deviation from the flat distribution suggests synchronization. In short, if $\triangle {\rm{\varphi }}({t}_{k})$(*k* = 1 … *N*) are the phase differences between two signals, PLI is computed by

1 $${\rm{PLI}}=\,|\langle {\rm{sign}}[{\rm{\Delta }}{\rm{\varphi }}({t}_{k})]\rangle |$$

where 〈〉 denotes mean operator. The range of PLI is between 0 and 1. A PLI of 1 means perfect phase locking at some value of $\triangle {\rm{\varphi }}$. And a PLI of 0 means either no coupling or coupling with a phase difference centered at 0 mod π. The larger PLI is, the stronger the phase locking will be.

The functional connectional analysis was performed as follows: the pre-processed EEG data was first subdivided into non-overlapping segments of length 5 s^35^; a 128 × 128 connectivity matrix was then derived for each segment by using PLI to calculate synchronization between all pair of channels; finally, to enhance the accuracy, an averaged connectivity matrix was obtained by taking into account all the connectivity matrix of each segments. All the graph analysis in the following was based on this averaged connectivity matrix.

### Analysis of network topology

**Figure 1:**
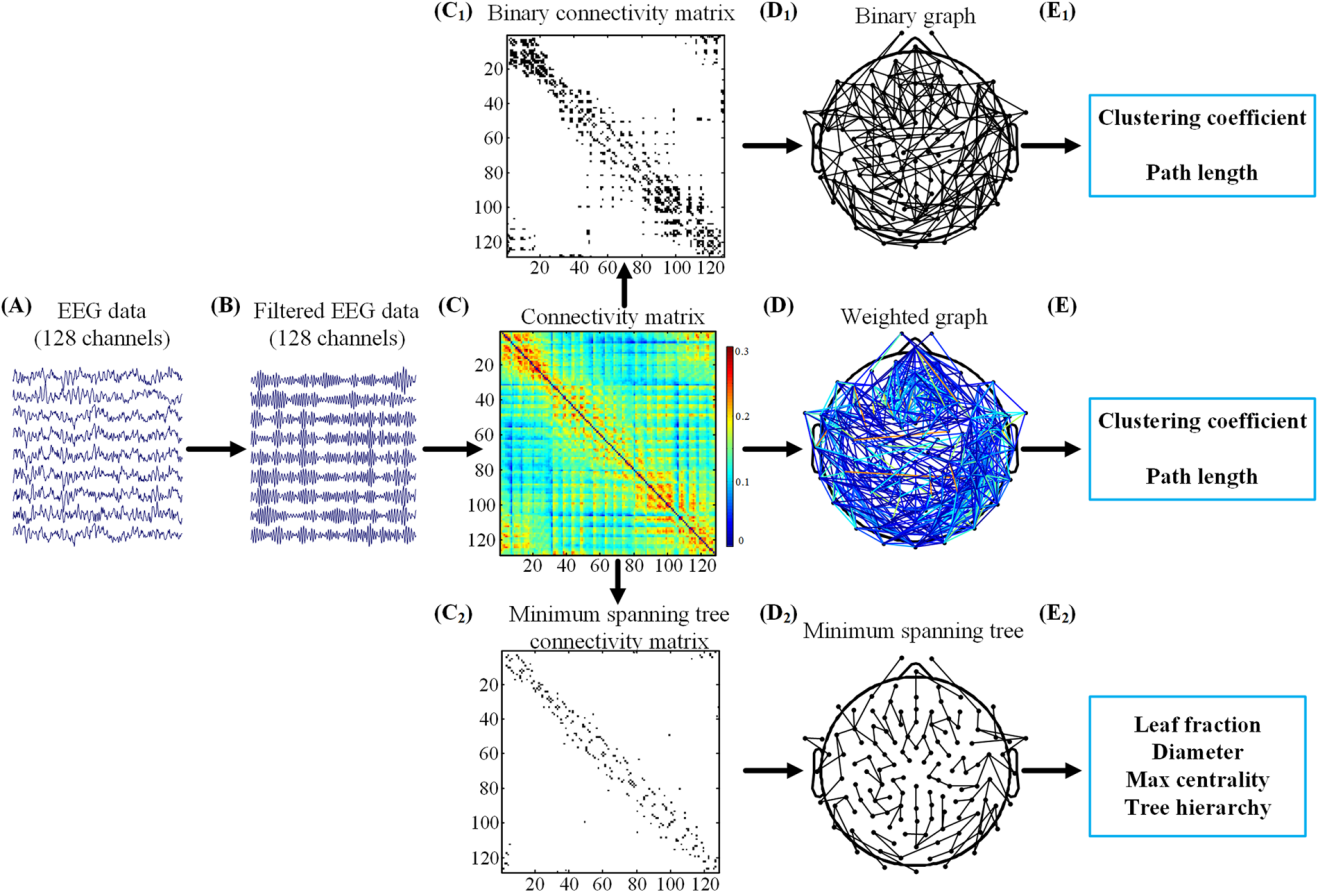
Schematic overview of graph analysis applied to EEG data using three different approaches: weighted graph, binary graph, and minimum spanning tree. After pre-processing (**A**), EEG data was filtered into the frequency bands of interest (**B**). Connectivity matrix (**C**) was constructed by using PLI to calculate synchronization between EEG channels. A weighted graph (**D**) was directly mapped from the connectivity matrix (**C**) for weighted graph analysis. For binary network analysis, a binary connectivity matrix (**C**_**1**_) only with strong connections was firstly converted from the connectivity matrix (**C**) by a threshold, and the corresponding binary graph (**D**_**1**_) was then mapped from the binary connection matrix. For MST analysis, connectivity matrix (**C**_**2**_) for the MST was first constructed from the original connectivity matrix (**C**) by Kruskal’s algorithm, and the corresponding MST (**D**_**2**_) was then mapped from the MST connection matrix. Finally, measures of network topology were computed for the weighted graph (**E**), the binary graph (**E**_**1**_), and the MST (**E**_**2**_) respectively.

The graph theory was adopted to examine the abnormal changes in the brain network topology of ASD. In general, graph can be represented by sets of nodes and edges between these nodes. If edge weight in a graph are either 1 (there is an edge between nodes) or 0 (there is no edge), the graph will be termed as binary graphs. And if edge weight in a graph are assign certain value to reflect the connection strength between nodes, the graph will be termed as weighted graphs. For a full investigation of the characteristics of brain network topology of ASD, both weighted and binary graph analysis were adopted in this study. Furthermore, the MST as another alternative method was used to characterize the brain network topology of ASD. Figure 1 gives an overview of our analysis framework, which included all steps involved in the computation of network measures for weighted/binary graph and MST.

### Weighted graph analysis

In the weighted graph analysis, we constructed a graph of 128 nodes (i.e. 128 electrodes) and directly used entities in the connectivity matrix as edge weights. A fully connected and undirected weighted graph can be seen in Fig. 1(D), in which the edge weight between nodes was assigned as synchronization strength between the corresponding EEG signals.

Topology features of weighted graph can be characterized by some network indexes. The clustering coefficient and average shortest path length are the two most fundamental measures, which have been widely used to analyze the brain function network. In the weighted graph, the clustering coefficient of a node indicates the proportion of its neighbors that are also connected with each other, and quantifies the tendency to form local clusters. The clustering coefficient of node *i* in weighted graph is defined as

2 $$C{C}_{i}=\frac{{\sum }_{k\ne i}{\sum }_{l\ne i,l\ne k}{w}_{ik}{w}_{il}{w}_{kl}}{{\sum }_{k\ne i}{\sum }_{l\ne i,l\ne k}{w}_{ik}{w}_{il}}$$

where *w* denotes the edge weight between two nodes. And clustering coefficient of the whole network can be calculated by

3 $${\rm{CC}}=\frac{1}{N}\sum _{i=1}^{N}C{C}_{i}.$$

The average path length reflects the efficiency of information transport in the network. It can be computed by the harmonic mean of the shortest path between all pairs of nodes in the graph^36^. In the weighted graph, the shortest path between two nodes is defined as the path with the largest total weight. In the practical computation, a connection-distance matrix, whose entries correspond to the “distance” between two nodes, is defined as the inverse of original connectivity matrix. Average weighted path length of the whole network can be calculated by

4 $${\rm{PL}}=\frac{1}{\frac{1}{N(N-1)}{\sum }_{i=1}^{N}{\sum }_{j\ne i}^{N}(1/{l}_{ij})}$$

where *l*_*ij*_ is the length of shortest path between node *i* and *j*.

From the definition, it should be noted that both values of CC and PL depend on network structure but also edge weight (connectivity values). To obtain measures that are independent of edge weight, clustering coefficient and path length are normalized by random networks. An ensemble of 100 surrogate random networks was derived from the original networks by randomly reshuffling the edge weights. Finally, the ratio between the weight and random clustering coefficient as well as the ratio between the weighted and random path length were computed as the normalized network measures.

### Binary graph analysis

Before binary graph analysis, a binary connection matrix (Fig. 1(C_1_)) should be converted from the original connection matrix, which was commonly performed by setting a certain absolute threshold. If some PLI value in the connection matrix exceeds the threshold, the corresponding entry in binary connection matrix was set as 1, otherwise set as 0. Then, a binary graph (Fig. 1(D_1_)) can be constructed based on the binary connection matrix, where an edge between two nodes exists if the responding entry in binary connection matrix is 1; otherwise no edge exists.

**Figure 2:**
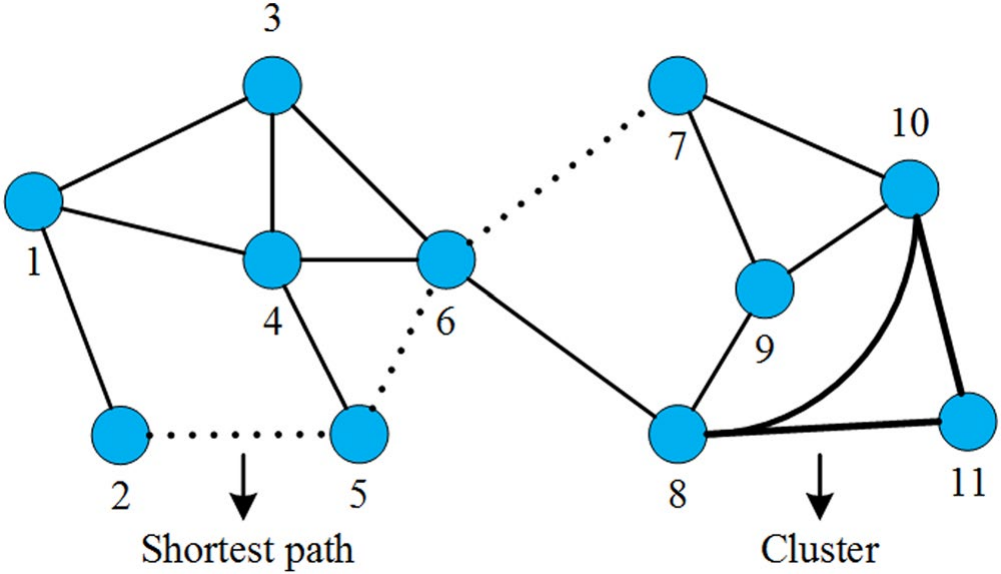
Measures of binary network topology. A cluster will form if the neighbors of a node are also directly connected, e.g., the bold triangle (node 8, 10, 11). Cluster coefficient of a node is defined as the fraction of existing edges between its neighbors. Shortest path is the path with minimum number of edges that connect two nodes, e.g., the dotted line between node 2 and 7.

Once the binary graph was constructed, clustering coefficient and average shortest path length can also be used to characterize its topology configuration. An illustration of cluster coefficients and path lengths in binary graph was shown in Fig. 2. The cluster coefficient of a node is defined as the fraction of existing edges among its neighbors. And the cluster coefficient of whole binary graph is the mean of clustering coefficient of all nodes. The average path length in binary graph can be computed by the mean of shortest path length between all pairs of nodes, and the shortest path between two nodes is defined as the path with minimum number of edges.

When the clustering coefficient and path length in the binary graph were computed at a given threshold, the results may be influenced by differences in the mean level of functional connection between ASD and TD. This is because the two network measures depend not only on the network topology structure, but also on the number of edges. Functional connections in ASD should be significantly lower than TD^37^, which means fewer edges in the binary graph of ASD at the given absolute threshold. To avoid this effect, the clustering coefficient and path length were computed here at certain link density, which refers to the fraction of retaining edges in the given size of graph. In this way, the binary graphs in both groups are guaranteed to have the same number of edges, so that any differences in network measures only reflect differences in topology of graph. Furthermore, as there is no unique way to choose an optimum link density, this study explored a range of link density, from 0.2 to 0.9 with increments of 0.05.

### Minimum spanning tree analysis

**Figure 3:**
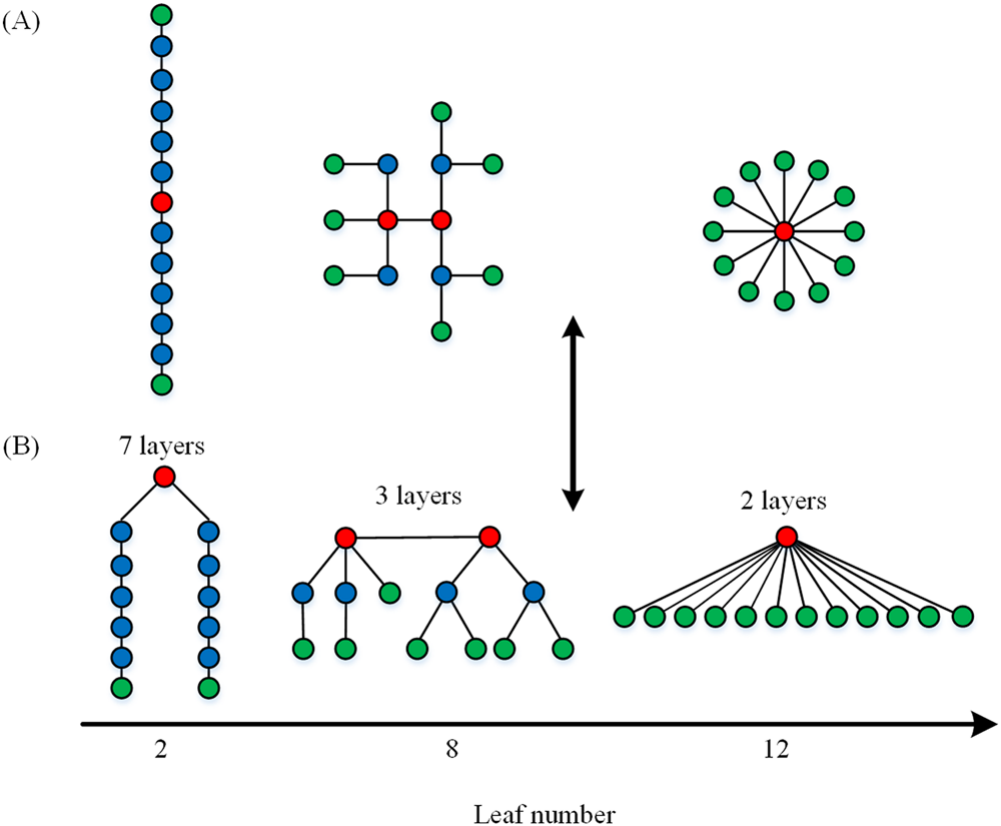
The topology and hierarchy of minimum spanning tree. For illustration, trees with different topology are depicted with increasing leaf number. All trees have thirteen nodes and twelve edges. Leaf nodes are depicted in green. Nodes with high centrality are displayed in red. And Nodes in blue don’t have special characteristics. (**A**) The left is a line-like tree with leaf number of 2, the right is a highly centralized star-like tree with leaf number 12, and the middle is corresponding to a tree with topology between the two extremes. Trees in (**B**) are identical to that in (**A**). With leaf number increasing, trees will have fewer layers.

The minimum spanning tree is defined as sub-network that connects all nodes without forming loops while minimizing the total link cost. The link cost in neuroimaging data should be considered as the inverse of connectivity strength, which means that the MST based on functional connectivity actually corresponds to a maximum spanning tree. In this study, Kruskal’s algorithm^38^ was adopted to constructed MST. After all weights of edges are ordered in an ascending way, the edge with the highest weight is first selected, and the consecutive edges with higher weight were then added until all *N* nodes were connected by *N*-1 edges in a loopless subgraph. When forming a loop, the edge added was skipped. In this study, a MST with 128 nodes and 127 edges was constructed as illustrated in Fig. 1(D_2_) and its corresponding connection matrix was shown in the Fig. 1(C_2_). There are two extreme topologies for MST, including a line-like tree with all nodes in a line and a highly centralized star-like tree with one central node, as shown in Fig. 3.

In MST analysis, several measures can be computed to describe its topological properties. Nodes with degree of 1 in a tree are called “leaves” or leaf nodes, and the number of leaves (LN) ranges from two (a line-like tree) to *N*-1 (a star-like tree). For comparison, MST leaf fraction is defined as the fraction of leaf nodes. The betweenness centrality (BC) is an indicator of centrality of a node, and can be used to reflect the relative importance of the node in the graph. The BC for a node is defined as the fraction of all shortest paths in the graph that go through this node. The range of BC is between 0 and 1, and a node with high BC is often referred as “hub” in the tree.

In general, the optimal MST should guarantee efficient communication between all nodes, such as MST with star-like topology. However, the central hub in a star-like MST is liable to overload, leading the whole network vulnerable. Hence, the optimal MST should have a good balance between large scale integration and overload prevention. To quantifying the level of balance, an indicator, called tree hierarchy (TH)^27^, was defined as

5 $${TH}=\frac{LN}{2{{BC}}_{{\max }}}$$

where *BC*_*max*_ is the maximum BC among all the nodes in MST. The range of TH is between 0 and 1. For a line-like MST, TH approaches 0 when node number approaches infinity. For a star-like MST, TH approaches 0.5. And TH will have a higher value for MST between these two extreme topologies.

### Statistical analysis

All analyses were performed using the SPSS Statistics 20.0 software package (IBM Corporation). Since almost all of network measures at each frequency band for each group were distributed normally (tested using a Shapiro–Wilk test), group differences were evaluated with independent *t*-test. As network measures in each graph methods may be correlated, Bonferroni correction was applied to *p* values for each frequency band in each graph analysis methods. In addition, in the binary graph analysis, there were 15 comparisons for each link density (from 0.2 to 0.9 with increments of 0.05), we also adopted Bonferroni correction for these comparisons. All p-values reported were corrected using the Bonferroni method, and the alpha significance level was set at 0.01 to balance the potential for type I and type II error in this study.

## Results

### Reduced functional connectivity in ASD

**Figure 4:**
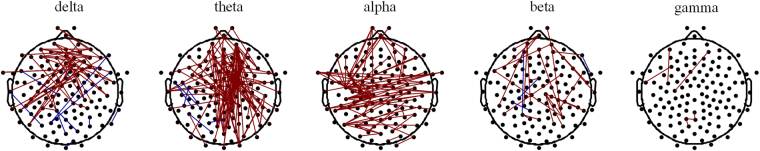
Topography of significantly different functional connection between TD and ASD in six frequency bands. Edges are drawn between channel pairs: red edges represent TD > ASD and the blue edges represent TD < ASD. Only edges that were significant different between groups at a false discovery rate corrected p value of 0.05 are plotted.

**Figure 5:**
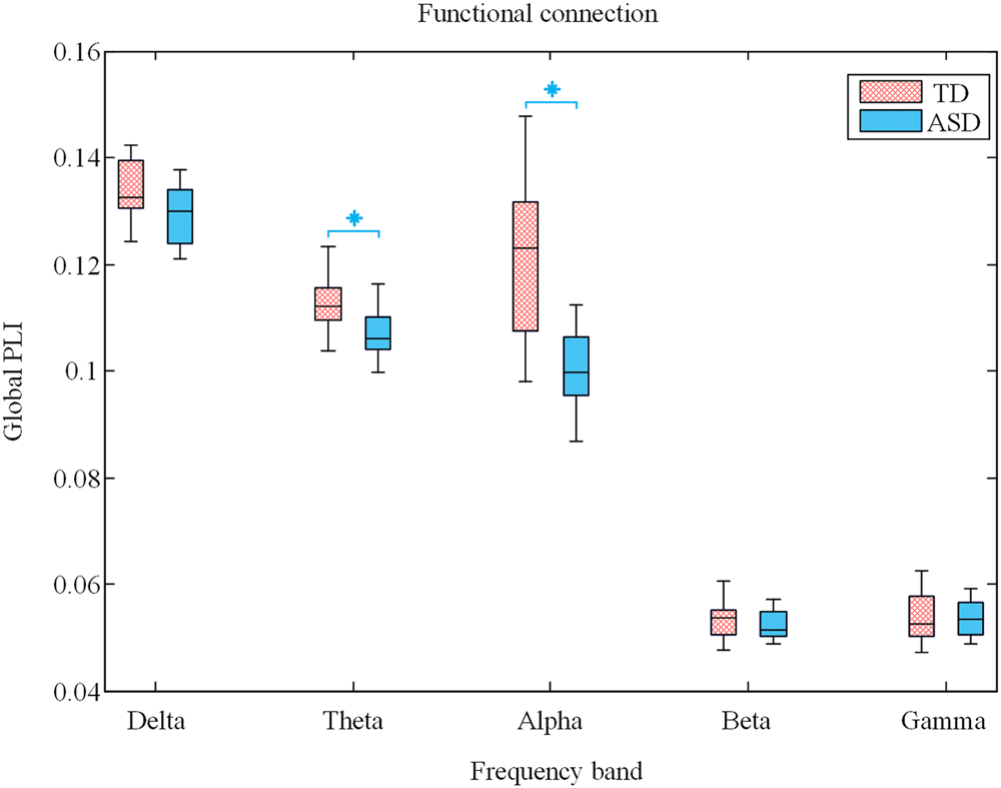
Mean PLI averaged over all pairs of EEG channels for TD (red) and ASD (blue) in five frequency band. Significant difference between TD and ASD with *t*-test (*p* < 0.01) are presented by blue asterisks (Bonferroni correction).

Figure 4 showed the topography of significantly different functional connectivity between TD and ASD in six frequency bands. From this figure, it can be intuitionisticly found that most of the atypical functional connectivity in ASD exhibited a decrease compared with the TD group, especially in delta, theta and alpha band. To quantify the trend, global mean functional connectivity was computed for all pair-wise combinations of channels in five frequency band respectively. As illustrated in Fig. 5, the mean PLI in ASD was significantly lower in the theta (*t* = 3.28, *p* = 0.002) and alpha band (*t* = 5.18, *p* < 0.001). There were no significant differences in other frequency band.

### Disrupted small-world topology in weighted brain network of ASD

**Figure 6:**
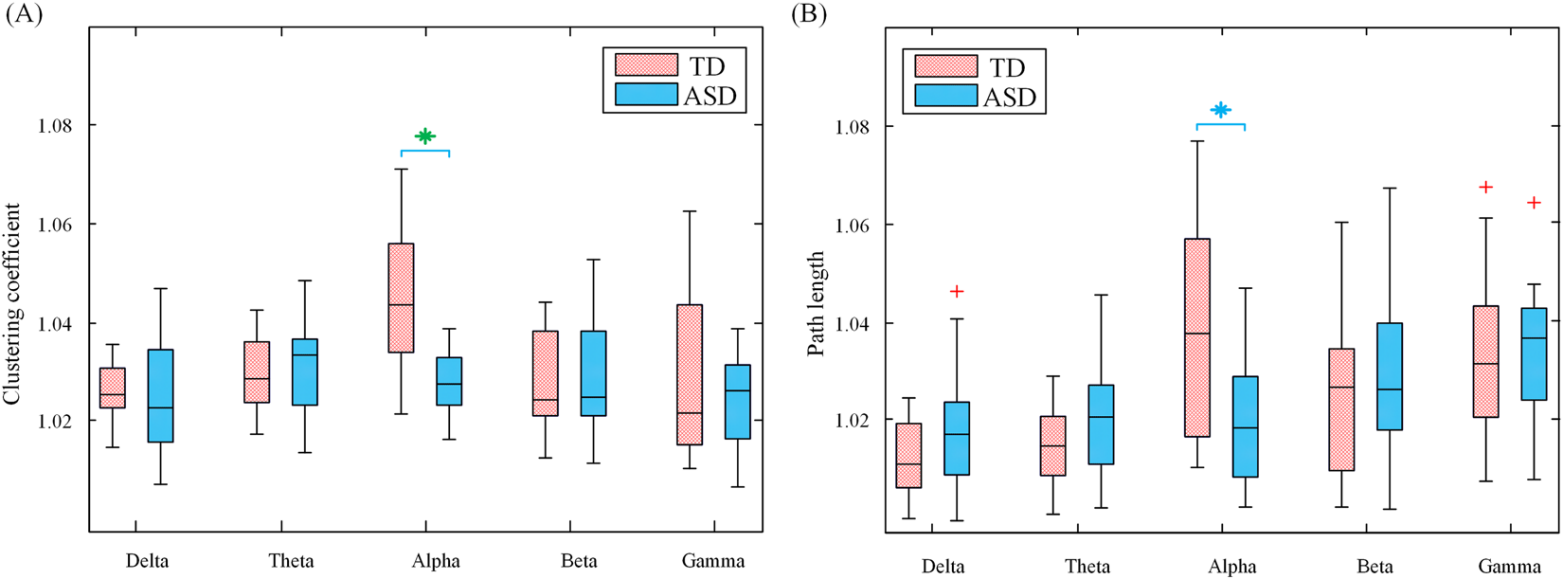
Boxplots showing clustering coefficient (**A**) and path length (**B**) for TD (red) and ASD (blue) groups at five frequency band in weighted graph analysis. Boxplot was depicted with first quartile, median, third quartile and outliers (red plus). Significant differences (*p* < 0.01) and marginally significant differences (0.01 < *p* < 0.05) were marked by blue and green asterisks respectively (Bonferroni correction).

In the weighted graph analysis, the clustering coefficient and path length were estimated for the brain function network of both TD and ASD groups in each frequency band. Boxplots of the clustering coefficient and path length were illustrated for the two groups in each frequency in Fig. 6(A) and (B) respectively. It can be observed that in the alpha band ASD group showed a significant decrease in normalized path length (*t* = 3.0, *p* = 0.009) together with a decrease trend in normalized clustering coefficient (*t* = 2.82, *p* = 0.016), and no significant difference was found in the other frequency bands. Taken together, these findings indicated the topology of brain network in ASD departed from a small-world network to a random network.

### Disrupted binary brain network of ASD for a range of link densities

In the binary graph analysis, the clustering coefficient and path length were calculated at a range of link density in each frequency band for both TD and ASD groups.

**Figure 7:**
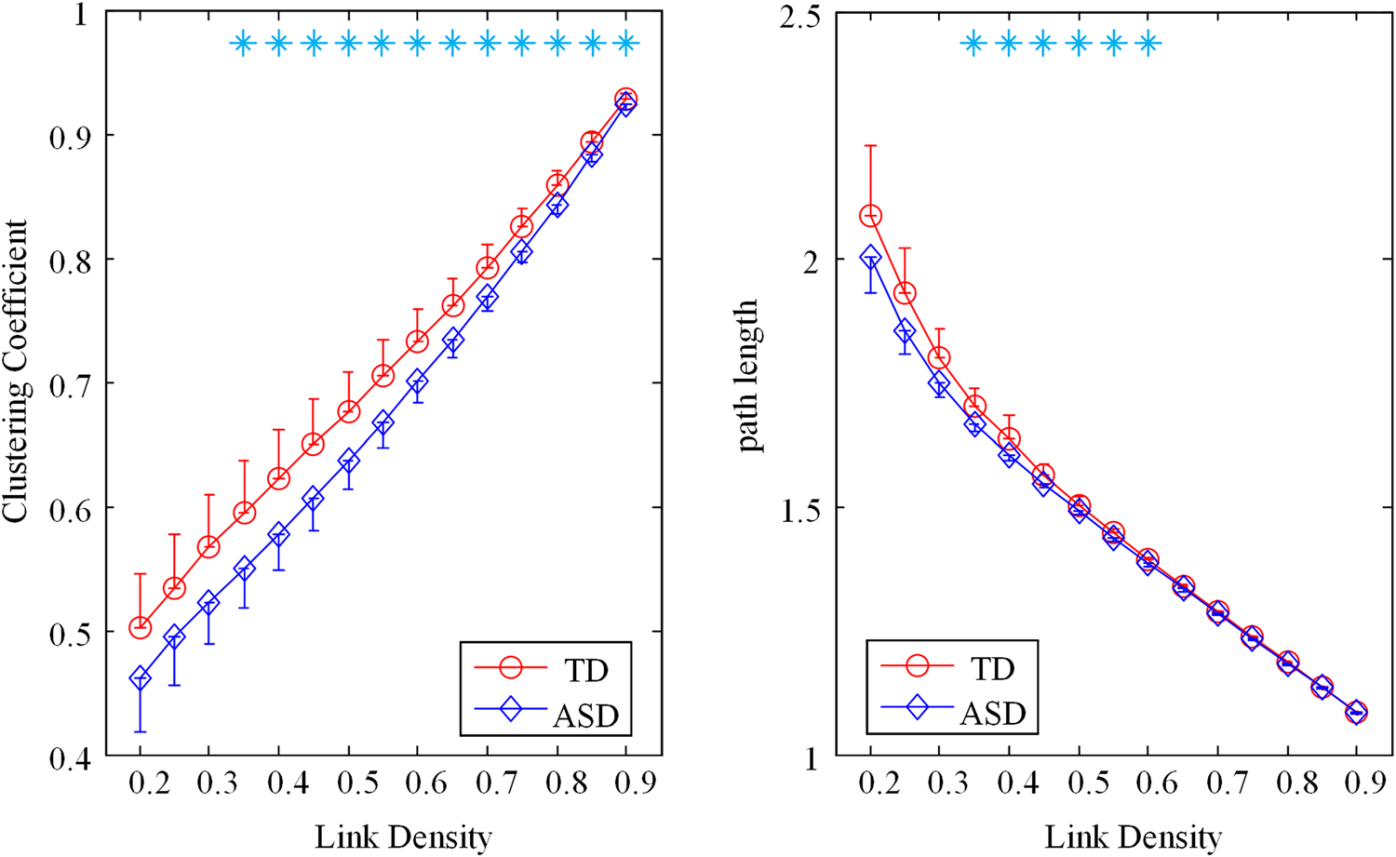
Cluster coefficient (**A**) and path length (**B**) as a function of link density for the TD (red circles) and the ASD group (blue diamonds) in the alpha band. Error bars are the standard error. Significant differences with independent *t*-test (*p* < 0.01) between the two groups are marked by blue asterisks (Bonferroni correction).

As illustrated in the Fig. 7(A), the clustering coefficient was plotted as a function of link density in the alpha band, and there was no difference in other frequency bands. For a high value of link density, the corresponding graphs were almost fully connected between all nodes yielding clustering coefficient close to 1 (for link density = 1, clustering coefficient was expected to be 1). With the decreasing of link density, more and more edges would be lost, and the corresponding cluster coefficient would also decrease. Over the whole range of link density investigated (from 0.2 to 0.9 with increments of 0.05), the clustering coefficient of ASD was consistently lower than the TD group, however, due to larger variance at lower link densities, the group differences were statistically significant only over the range from 0.35 to 0.9.

The path length was plotted as a function of link density for alpha band in the Fig. 7(B), and no difference was observed in the other frequency bands. With the increasing link density, more edges would be added, resulting in the decreasing path length. For low link density (0.2–0.3), path length of ASD was slightly lower than that of TD, but no statistical differences due to the large variance. For intermediate range of link density (0.35–0.6), the path length in ASD were significantly lower than the TD. For high link density (0.65–0.9), there was no difference between these two groups.

### A Line-like MST in ASD

**Figure 8:**
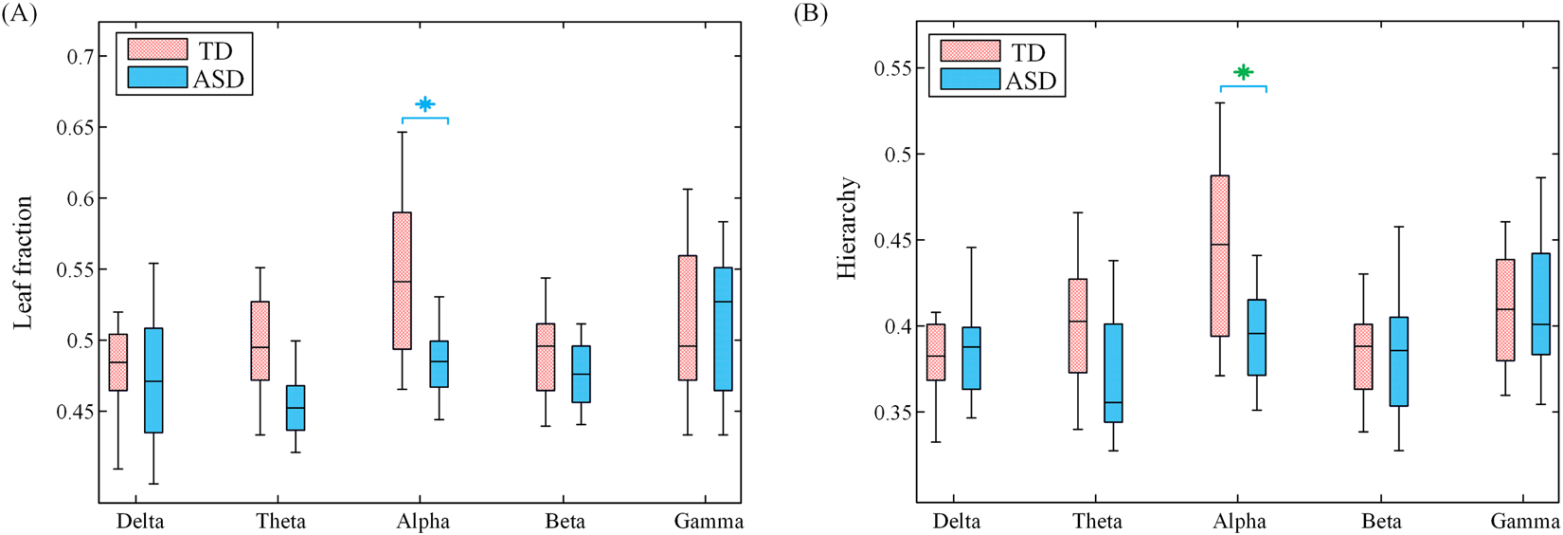
Boxplots showing leaf fraction (**A**) and tree hierarchy (**B**) for TD (red) and ASD (yellow) groups at five frequency bands in MST analysis. Significant differences (*p* < 0.01) and marginally significant differences (0.01 < *p* < 0.05) were marked by blue and green asterisks respectively (Bonferroni correction).

**Figure 9:**
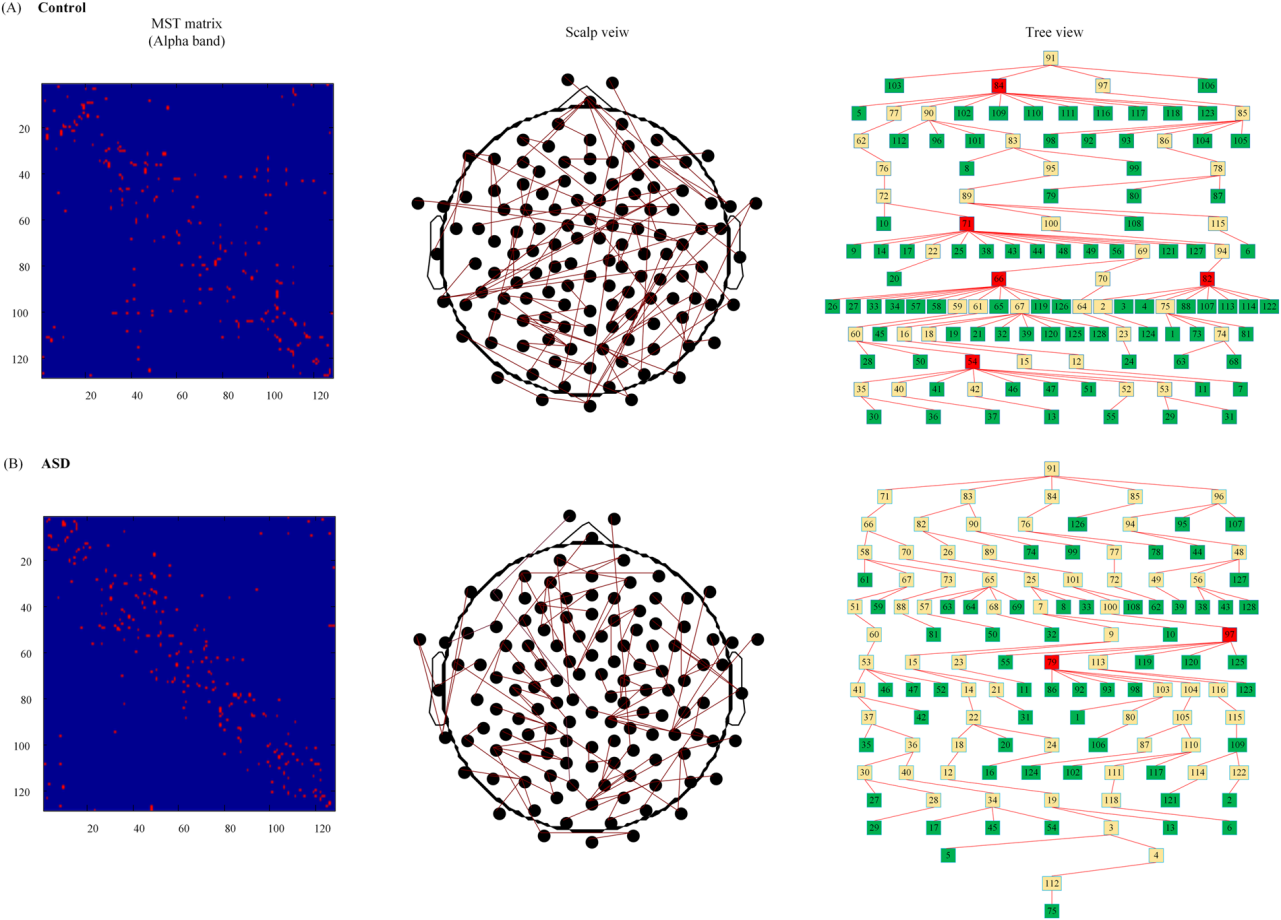
MST matrices (left panels) and MST graph in scalp view (central panels) and tree view (right panels) at the alpha band for controls (**A**) and ASD (**B**). For illustrative purpose the MST algorithm was performed on the averaged connectivity matrix.

Boxplots of the leaf fraction and tree hierarchy were illustrated for TD and ASD groups in Fig. 8(A) and (B) respectively. MST analysis yielded significant group effects in alpha band (see Figs 8 and 9). Leaf fraction, reflecting the integration of information within the network, was significantly lower in ASD group relative to TD group (*t* = 3.4, *p* = 0.006). The group effect on tree hierarchy just fell short of significance (*t* = 2.61, *p* = 0.029), suggesting a trend for a loss of balance between large scale integration and overload prevention in ASD compared to TD group. Collectively these results indicate a shift towards a more line-like tree configuration in the brain network of ASD.

## Discussion

Autism is a complex neurodevelopmental disorder that affects multiple cognitive domains and brain systems. Questions regarding the strength (over-connectivity or under-connectivity), space (widespread or focal), and developmental timeframe (childhood or adulthood) of aberrant brain connectivity in autism are hotly debated. In this study, we attempted to investigate how the brain networks changes in children with ASD by using functional connectivity analysis and graph analysis including the commonly used weighted and binary graph, as well as minimum spanning tree. We found that childhood autism is characterized by a disrupted brain networks, which may be a distinguishing feature in children with ASD.

The whole brain functional connectivity in ASD was found to be significant reduced, which suggested an overall under-connectivity of functional brain networks in ASD. And the overall decrease connectivity in alpha band aligned well with the decrease of alpha power during resting state in ASD previous reported by Wang^6^. Recently, Kikuchi *et al*. employed a custom children-size MEG system and focused on the theta band neural connectivity to investigate the aberrant long-range networks in ASD^39^. Their results showed that ASD group exhibited a significant decrease in the coherence of theta band compared with TD group, which were also consistently with our functional connectivity results in theta band. In addition, a combined fMRI and diffusion-weighted imaging examined the functional and structural connectivity of children with ASD and also found overall reduced functional connectivity between distributed imitation regions in the ASD group^40^. However, recent theoretical and empirical work is beginning to reveal that autism is associated with a complex functional phenotype characterized by both hypo- and hyper-connectivity of large-scale brain systems^14^. This discrepancy might be because the autisms in this study were children and development changes should be taken into account^41^. Nevertheless, the current study supported the opinion that the children with ASD can be viewed as a disconnection syndrome^42,43^, and the functional connectivity analysis may be a promising method to reveal the abnormalities associated with ASD.

The classic autistic cognitive profile of superior simple information processing and impaired higher order information processing stresses the importance of studying brain networks as a whole rather than only specific connections^44^. The graph theory provides a way to examine the alternations in functional connectivity at a higher and integrative network topology level. Both weighted and binary graph analysis found decreased clustering coefficient and path length in ASD, suggesting a disrupted segregation and integration organization in their brain networks. Based on the definition of small-world network, such disrupted network topology meant that the brain networks in ASD departed from small-world network to random network. Actually, such kind of the network deterioration has been found in adults with ASD^45,46^; however, our study extends the findings by evidencing that such abnormalities are already present in children with ASD. During normal development in children, studies have found that the brain maturation processes occur with pruning redundant connectivity and strengthening core connectivity to shape the whole network into a more efficient organization (i.e., highly interconnected networks with low cost)^17,47^. It is suggested that brain functional networks in children shift from a random toward a more organized small-world configuration with maturation^48^. On the contrary, the current study found that the brain networks in children with ASD shifted to a random architecture, which may be explained by an impaired pruning of connections in the developmental process.

A major strength of our study is the application of MST as a reliable and fully unbiased graph theoretical approach in addition to conventional approaches. In MST analysis, leaf fraction in ASD was lower than TD, indicating a less integrated brain networks of ASD. Furthermore, the lower leaf fraction also suggested that brain networks in ASD was more line-like, decentralized organization compared to controls. A study about life-span development from general populations indicated that a shift from a line to a more star-like network configuration occurs from childhood through early adulthood^49^. Hence, this distinct brain network topology may be associated to the pathology mechanism of ASD. In addition, a lower tree hierarchy was found in MST of ASD, meaning that the balance between large scale integration and overload prevention was disturbed in brain networks of ASD. As tree hierarchy quantified the extent to which a MST display the optimal organization, the decreased tree hierarchy in MST indicated a less optimal organization in brain networks of ASD. A lower hierarchical structure has shown to be associated with poorer cognitive performance^50^, together our findings indicated that MST may increase our understanding to the neurobiology of ASD. In all, our work evidenced that MST offered an elegant unbiased method to capture subtle variations in the organization of brain networks, and can be used to distinguish pathological networks in ASD from healthy normal networks in TD.

The primary rationale for using MSTs for brain network topology analyses is the fact that the computed network measures are not biased by network size, and density effects. This is of particular importance, as there is currently no normalization step that allows for a reliable and fully unbiased characterization of network topology. When using weighted or binary graph analysis, differences in connectivity strength, graph size and connectivity density within or between subjects may therefore yield spurious findings or mask true effects. Moreover, MST tends to constitute the most important connections or the ‘information highways’ of the whole brain network. This implies the probability of MST containing the set of shortest paths is high and the MST may be considered as the critical backbones of the original networks. Hence, the observed group differences between ASD and TD would correspond to alternations in critical backbone of brain networks rather than irrelevant or less important connectivity. On the other hand, some relevant information about the brain networks may be inevitably lost in MST when not all, but only the ‘core’ connections are taken into account. For example, local segregation such as clustering is not as easy to inspect in MST as in conventional graph analysis. Overall, MST network analyses cannot be considered as a total substitute for conventional analyses but for many questions or issues it offers an elegant unbiased method to compare networks. Future methodological studies in healthy controls and modelling studies are needed to further elucidate how analysis of MST can be used to increase our understanding of the organization of functional brain networks.

Interestingly, our findings tended to indicate that brain activity in theta and alpha band was disturbed in children with ASD, as suggested by the observation that abnormal changes of brain networks concentrated in these two frequency bands. As the synchronization in theta and alpha band have been proved to be greatly involved in large scale integration of cortical information^51^, we can say that the disrupted brain networks in theta and alpha band meant large-scale changes in the brain functional network of ASD. Indeed, many previous studies about ASD have reported abnormality in the theta and alpha band^6,52^. In our previous work, we applied EEG to monitor outcomes of a prefrontal neurofeedback treatment for individuals with ASD, and found a linear decrease of theta/beta ratio over 18 weekly sessions of neurofeedback, as well as improved performance on an attention test^53^. Gabard-Durnam *et al*. found different patterns of alpha asymmetry between ASD high-risk and low-risk infant populations, supporting the candidacy of alpha asymmetry as an early neural ASD endophenotype^54^. With a custom child-sized MEG system, Kikuchi *et al*. found that childhood autism displayed significantly reduced functional connectivity between the left and right posterior areas in theta band^39^. Furthermore, studies about brain development stressed brain activity in alpha frequency band was vitally important for brain maturation^20^. Hence, our findings indicated disturbed brain activity in the theta and alpha bands in ASD patients, which may be involved in the neurobiological origins of ASD.

Several limitations in this study also should be mentioned. First, the sample size in this study is not sufficiently large to draw any definite conclusions concerning the distinction between the ASD and TD in brain functional network. Larger prospective studies are necessary to verify the findings in this study. Nevertheless, our results provide the evidence for a disrupted network topology in children with ASD. Second, the ASD participants studied in this work were high-functioning children from 7 to 13 years old, it is unknown how the results may generalize to individuals of other ages or intellectual levels. As abnormal brain development in autistic toddlers has been reported, investigating the brain networks topology at the early developmental stages may be more beneficial to understand the pathology of ASD. Finally, we are also agnostic about whether the observed changes in brain networks topology are specific to ASD. Further clarity would become available when similar studies are conducted on other neurodevelopmental conditions, such as intellectual disability and attention-deficit/hyperactivity disorder.

In conclusion, the present study demonstrated abnormal functional network in children with ASD, indicating that affected brain network in autism may already present at the early stages of brain development. The whole brain connectivity in ASD was found to be significantly reduced, supporting the concepts of ASD as disconnection syndrome. And the findings with conventional graph analysis methods revealed an aberrant organization in the brain functional network, suggesting a shift from small-world architecture to random network in ASD. Furthermore, the current study with MST found a decentralized and less optimal configuration in ASD. Taken together, these findings support the interpretation that not merely connectivity strength was affected in children with ASD but also involves an aberrant organization of connectivity. Hence, the disrupted brain networks in ASD may offer insight into the underlying pathology and possible serve as a biological marker that may aid in the diagnosis of this condition.
